# TREM-1, HMGB1 and RAGE in the Shoulder Tendon: Dual Mechanisms for Inflammation Based on the Coincidence of Glenohumeral Arthritis

**DOI:** 10.1371/journal.pone.0165492

**Published:** 2016-10-28

**Authors:** Finosh G. Thankam, Matthew F. Dilisio, Nicholas E. Dietz, Devendra K. Agrawal

**Affiliations:** 1 Department of Clinical & Translational Science Creighton University School of Medicine, Omaha, NE, United States of America; 2 Department of Orthopedic Surgery Creighton University School of Medicine, Omaha, NE, United States of America; 3 Department of Pathology Creighton University School of Medicine, Omaha, NE, United States of America; Pennsylvania State Hershey College of Medicine, UNITED STATES

## Abstract

Rotator cuff injury (RCI) is a major musculoskeletal disorder in the adult population where inflammation and pain are major contributing factors. Coincidence of other clinical conditions like glenohumeral arthritis aggravates inflammation and delays the healing response. The mechanism and signaling factors underlying the sustenance of inflammation in the rotator cuff joint are largely unknown. The present article aims to elucidate the involvement of inflammatory molecule, TREM-1 (Triggering Receptors Expressed on Myeloid cells-1), and danger-associated molecular patterns (DAMPs), including high mobility group protein 1 (HMGB-1) and RAGE (receptor for advanced glycation end products), in the setting of RCI with respect to the severity of glenohumeral arthritis. Biceps tendons (15 specimens) from the shoulder and blood (11 samples) from patients with glenohumeral arthritis (Group-1, n = 4) and without glenohumeral arthritis (Group-2, n = 11) after RCI surgery were obtained for the study. Molecular and morphological alterations between the groups were compared using histology, immunofluorescence, RT-PCR and flow cytometry. MRI and histomorphology assessment revealed severe inflammation in Group-1 patients while in Group-2 ECM disorganization was prominent without any hallmarks of inflammation. A significant increase in TREM-1 expression in circulating neutrophils and monocytes was observed. Elevated levels of TREM-1, HMGB-1 and RAGE in Group-1 patients along with CD68^+^ and CD16^+^ cells confirmed DAMP-mediated inflammation. Expression of TREM-1 in the tendon of Group-2 patients even in the absence of immune cells presented a new population of TREM-expressing cells that were confirmed by real-time PCR analysis and immunofluorescence. Expression of HMGB-1 and RAGE in the biceps tendon from the shoulder of patients without glenohumeral arthritis implied TREM-1-mediated inflammation without involving immune cells, whereas in patients with glenohumeral arthritis, infiltration and the activation of the immune cells, primarily macrophages, release mediators to induce inflammation. This could be the reason for ECM disorganization without the classical signs of inflammation in patients without glenohumeral arthritis.

## Introduction

Musculoskeletal disease is the leading cause of disability and healthcare cost in the United States [1, 2] and account for almost 20% of all healthcare visits. The shoulder is the second most common site of joint pain in patients that suffer from chronic musculoskeletal disease. The most common causes of shoulder pain in the adult population are rotator cuff injuries (RCI) and glenohumeral arthritis. Inflammation associated with RCI and arthritis is a major factor in the pathogenesis of tendon function [3]. The persistence of inflammatory cells, proinflammatory cytokines and angiogenesis in injured rotator cuff (RC) tendon induce fibrotic changes resulting in scarring [4]. Decreasing inflammation is one of the main strategies for treating patients with rotator cuff injuries and/or arthritis. However, the etiological factors associated with the regulation of inflammation in RC tendon are largely unknown.

TREM-1 (Triggering Receptors Expressed on Myeloid cells-1) is an immunoglobulin like superfamily receptor (expressed predominantly on immune cells) that upregulates inflammation [2, 5]. Soluble form of TREM-1 (sTREM-1) acts as a TREM-1 antagonist and downregulates inflammation [6]. sTREM-1 levels increase during inflammatory conditions such as arthritis, and are associated with the severity of inflammatory diseases [1, 7]. Another form of TREM, the TREM-2, in most cases, possesses an anti-inflammatory function [8, 9]. High mobility group box protein 1 (HMGB-1) and RAGE (the receptor for advanced glycation end products) trigger inflammation by enhancing cytokine secretion from immune cells at the injury site [10–14] and bind to TREM-1, but their exact role, interplay, and mechanism in the inflammatory pathway are unknown. While symptomatic osteoarthritis is associated with an elevated level of acute phase reactants in the blood [13, 15, 16] the role of TREM-1, HMGB-1, and RAGE are yet to be defined in musculoskeletal pathology.

An effective approach to treat most musculoskeletal diseases is to decrease inflammation through anti-inflammatory medications, which is usually in the form of corticosteroids or non-steroidal anti-inflammatory drugs (NSAIDs). Because TREM-1, HMGB-1, and RAGE affect inflammation in pathologic musculoskeletal conditions such as osteoarthritis, pharmacological manipulation of these mediators may represent a novel therapeutic approach to treat a variety of debilitating diseases. The purpose of this study is to investigate the role of TREM-1, HMGB-1, and RAGE in shoulder tendon tissues and systemic circulation in the setting of glenohumeral arthritis. Our hypothesis is that the severity of the musculoskeletal pathology directly correlates with the levels of these inflammatory mediators.

## Results

### Enhanced features of ECM disorganization, cellularity and angiogenesis in the shoulder tendon of the arthritis compared to non-arthritis group

**Fig 1:**
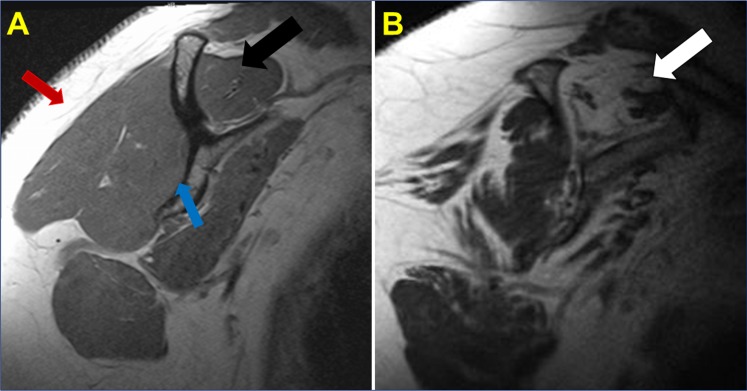
(A) T1 Sagittal magnetic resonance imaging (MRI) image of healthy rotator cuff musculature. Normal muscle appears dark on a T1 sequence, in contrast to the subcutaneous fat that appears light (white arrow). The black arrow denotes the healthy supraspinatus muscle belly without atrophy or fatty infiltration. The blue arrow points towards the body of the scapula. (B) Sagittal oblique T1 image of the shoulder in a patient with a chronic, massive rotator cuff tear. In contrast, T1 Sagittal MRI image of the shoulder demonstrates significant fatty infiltration and atrophy of the supraspinatus in the setting of a chronic, massive rotator cuff tear. Note the fatty infiltration of the supraspinatus tendon (white arrow) where the muscle has a similar white radiographic appearance as the subcutaneous fatty tissue (red arrow).

**Fig 2:**
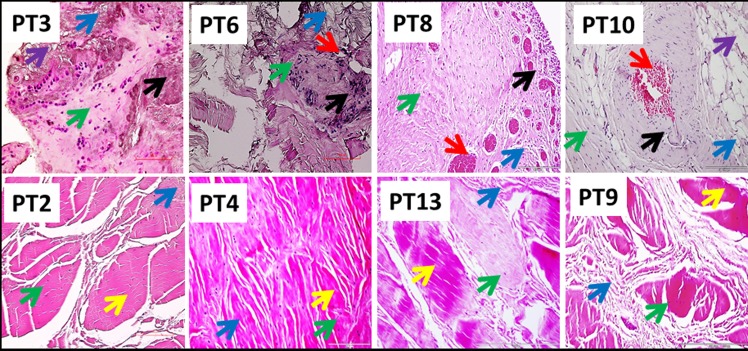
H&E staining of biceps tendons—Group-1 (Patient numbers—PT3, PT6, PT8 and PT10) and Group-2 (Patient numbers–PT2, PT4, PT13 and PT9). The green arrows show tendon cells; black arrows point inflammation; red arrows indicate angiogenesis; blue arrows show ECM disorganization; violet arrows point fatty infiltration; and yellow arrows indicate normal ECM with dense collagen deposition. The inflammation and fatty infiltration were not evident in Group-2 while ECM disorganization was less prominent compared to Group-1. The figures are shown in 400x magnification.

Patients were categorized into two groups; Group-1 consists of 4 patients with severe arthritis and a rotator cuff tear and Group-2 included 11 patients with a superior labral anterior to posterior (SLAP) tear without arthritis or a full thickness rotator cuff tear. Histomorphological changes, as determined by H&E staining (qualitatively), revealed altered physiological architecture of tendon tissue in all patients with disorganized extracellular matrix. The inflammation in the tendon and supraspinatus muscle was prominent in Group-1 while the inflammation was very mild/negligible in Group-2 as assessed by MRI and histology analysis. The presence of immune cells was evident in Group-1 while negligible in Group-2. The normal tendon cells of Group-2 patients were characterized by their less dense distribution of nuclei in the intact ECM. Out of the 4 patients in Group-1, supraspinatus muscle of the two showed significant fatty infiltration as evident in MRI (**Fig 1**). Similarly, Group-1 patients exhibited extensive neoangiogenesis in the tendon tissue that was not present in Group-2. The ECM disorganization in the tendon tissue was present in both groups, but most prominent in Group-1. Clustering of tenocytes was evident in the vicinity of ECM disorganization in Group-2. The results are shown in **Fig 2**.

### Increased expression of TREM-1 and TREM-2 in circulating CD16+ neutrophils and CD14+ monocytes of arthritis compared to non-arthritis group

**Fig 3:**
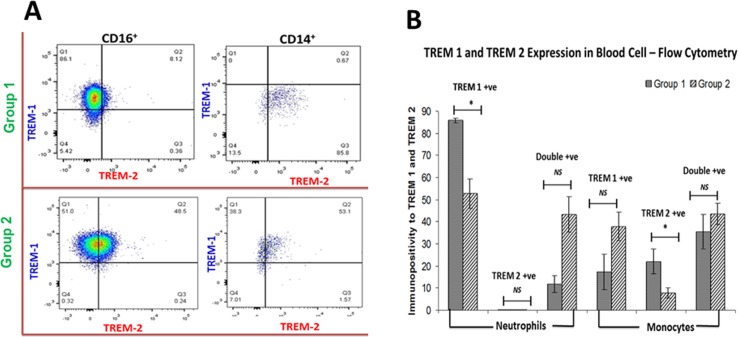
Flow cytometry analysis of TREM-1 and TREM-2 expression on CD16^+^ and CD14^+^ cells. (A) Representative images for the gating of the cells in Group-1 and Group-2 patients. (B) Comparison between Group-1 and Group-2 patients showing increased TREM-1 expression in the cells of Group-1 revealing the severity of inflammation. *n = 8 for both the groups*, * p<0.*05; NS–Not significant*

TREM expression in circulating immune cells was determined by flow cytometric analysis (**Fig 3A and 3B**). TREM-1 expression in neutrophils was significantly higher in Group-1 patients than in Group-2. TREM-2 expression in CD14^+^ cells was also greater in Group-1 than in Group-2. TREM-2 expression in neutrophils in Group-1 was negligible. There was a trend of increase, but statistically insignificant, in TREM-1 and TREM-2 double-positive cells (for CD14^+^ *P* value = 0.317 and for CD16^+^ *P* value = 0.182) in the blood of Group-2 (n = 8) than in Group-1 (n = 3).

### TREM-1 and TREM-2 expression in tenocytes and increased expression of HMGB1 and RAGE in the shoulder tendon of arthritis compared to non-arthritis group

**Fig 4:**
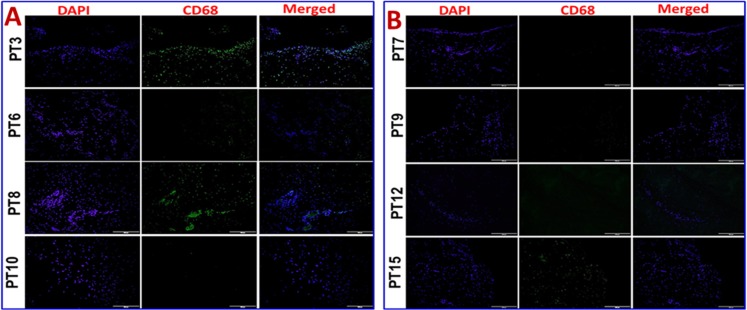
Immunofluorescence analysis for CD68^+^expression in the tendon tissues of Group-1 and Group-2 (A) Group-1 (Patient numbers—PT3, PT6, PT8 and PT10) and (B) Group-2 (Patient numbers–PT17, PT9, PT12 and PT15) patients. Group-1 tendons have significantly higher density of macrophages whereas in Group-2 the number of macrophages was negligible.

**Fig 5:**
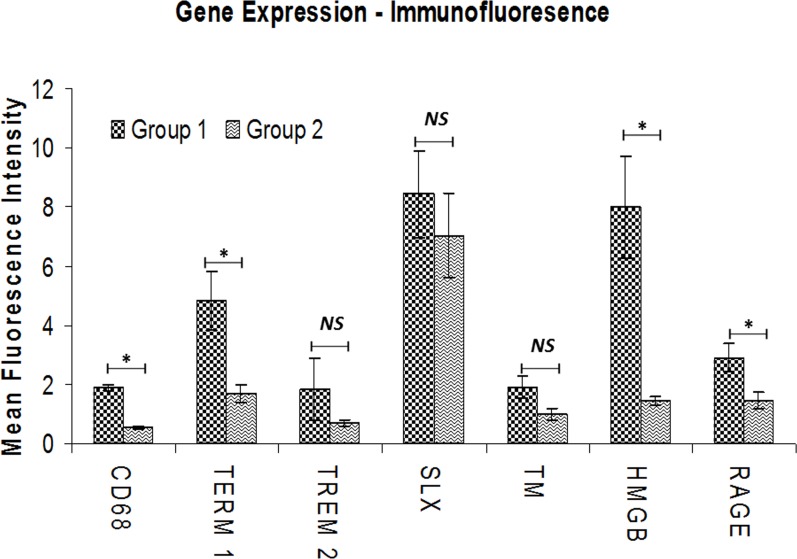
Mean fluorescence intensity for the evaluation of gene expression analysis by immunofluorescence using ImageJ software. The expression levels of CD68, TREM-1, HMGB1 and RAGE were significantly higher in the tissues of Group-1 when compared to Group-2. TREM-2 expression was enhanced in Group-1 but was not significant. The expression of TM and SLX between both the groups was mostly similar and not significant. *Group-1 (n = 4)*, Group-1 (n = 11) * p<0.*05; NS–Not significant*.

**Fig 6:**
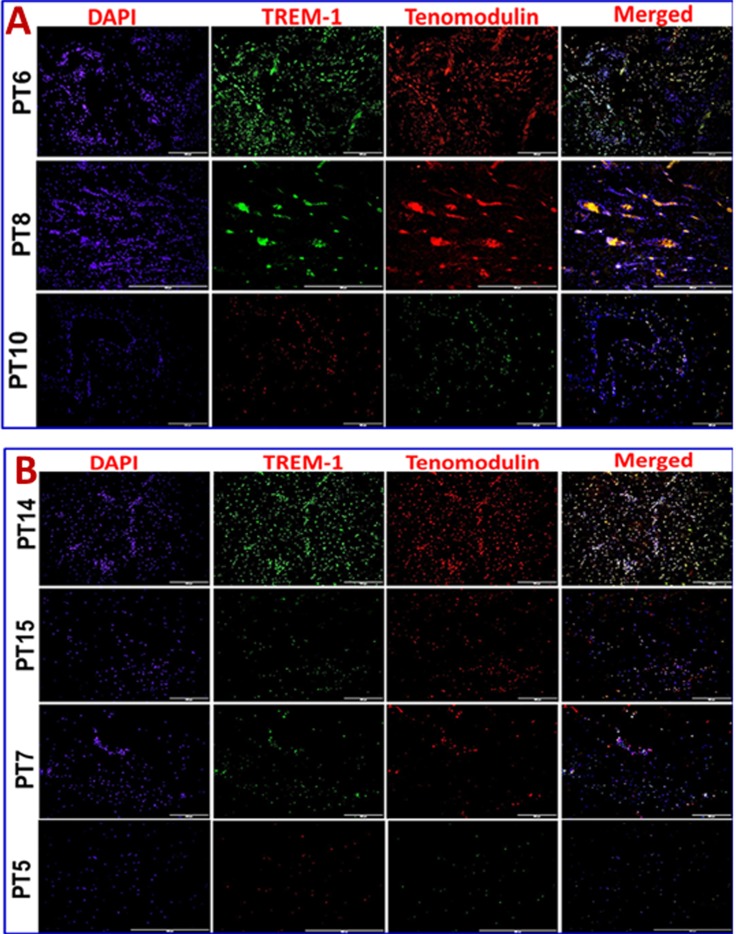
Double immunostaining for the co-localization of TREM-1 expression with tenomodulin (TM) on Group-1 (Patient numbers—PT6, PT8 and PT10) and Group-2 (Patient numbers–PT14, PT15, PT7 and PT5) tendons—(A) Group-1 and (B) Group-2 patients. Group-1 tendons have significantly higher TREM-1 expression whereas in Group-2 TREM-1 expression was lesser but prominent. TM expression was similar in the tendon tissue of both groups.

**Fig 7:**
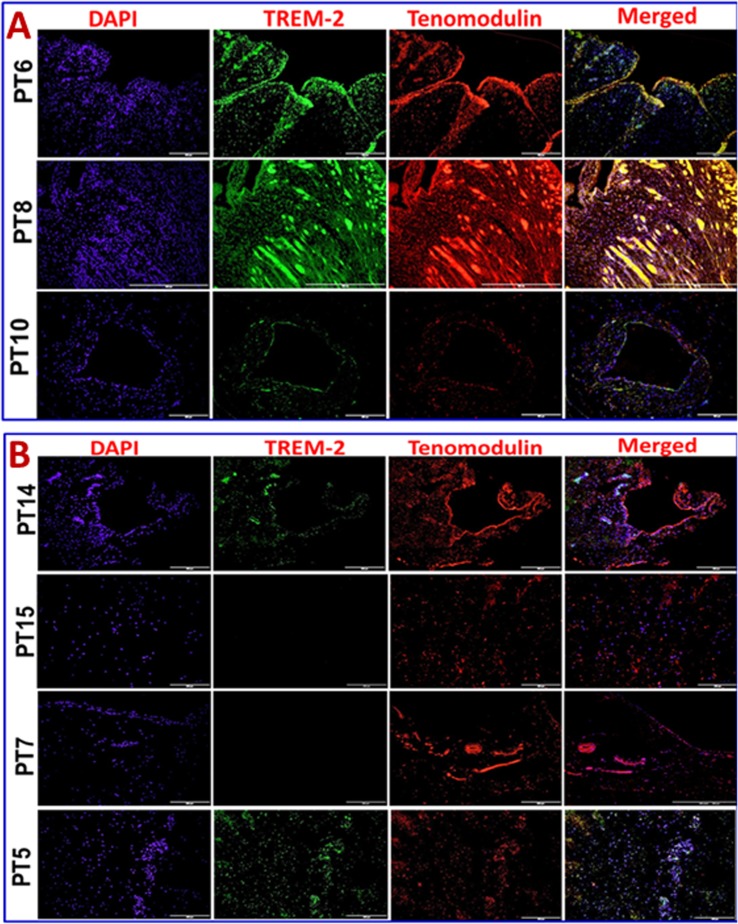
Double immunostaining for the co-localization of TREM-2 expression with tenomodulin (TM) on Group-1 (Patient numbers—PT6, PT8 and PT10) and Group-2 (Patient numbers–PT14, PT15, PT7 and PT5) tendons—(A) Group-1 and (B) Group-2 patients. Group-1 tendons have slightly higher TREM-2 expression when compared to Group-2. TM expression was similar in both the groups.

**Fig 8:**
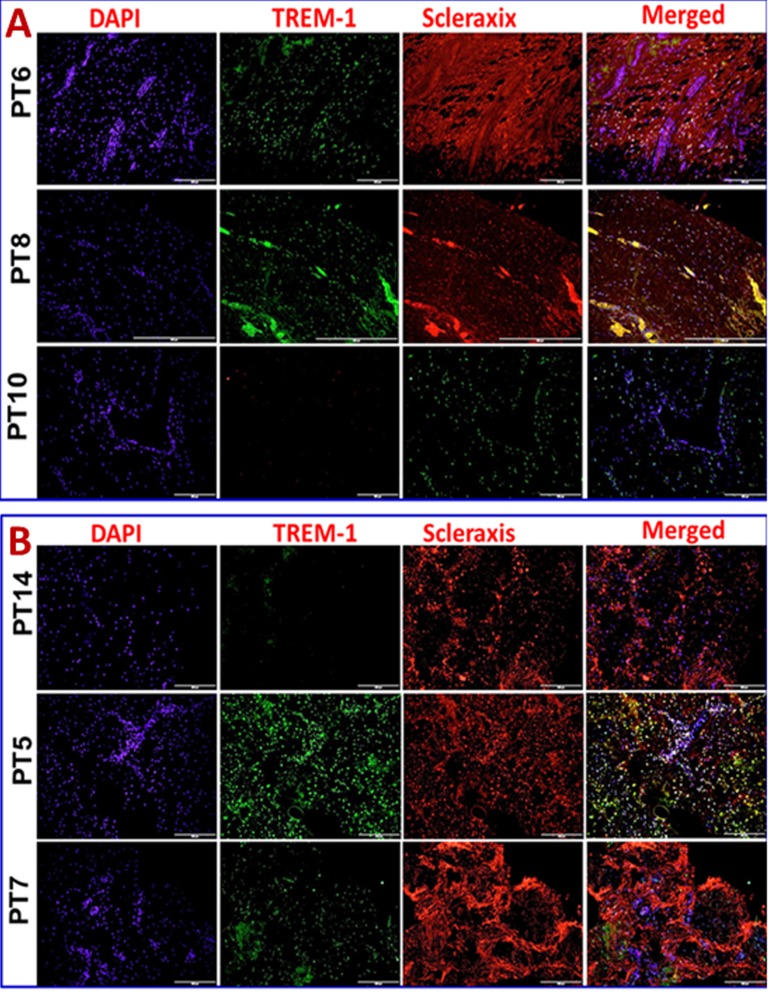
Double immunostaining for the co-localization of TREM-1 expression with scleraxis (SLX) on Group-1 and (Patient numbers—PT6, PT8 and PT10) and Group-2 (Patient numbers–PT14, PT5 and PT7) tendons—(A) Group-1 and (B) Group-2 patients. Group-1 tendons have significantly higher TREM-1 expression whereas in Group-2 TREM-1 expression was lesser but prominent. SLX expression was similar in both the groups.

**Fig 9:**
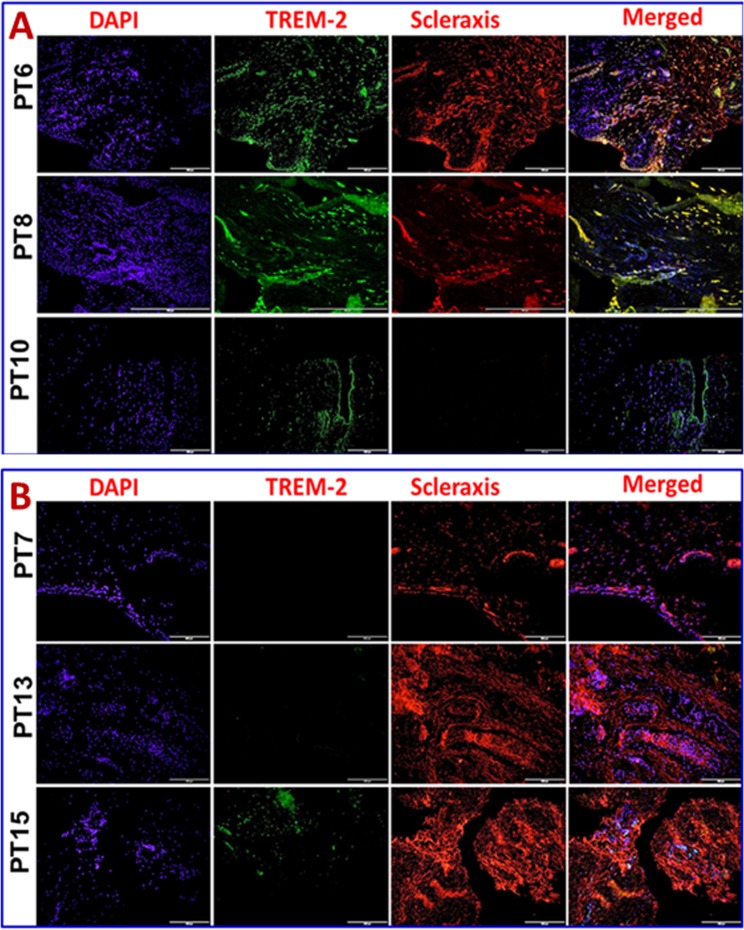
Double immunostaining for the co-localization of TREM-2 expression with scleraxis (SLX) on Group-1 ((Patient numbers—PT6, PT8 and PT10)) and Group-2 (Patient numbers–PT7, PT13 and PT15) tendons—(A) Group-1 and (B) Group-2 patients. Group-1 tendons have significantly higher TREM-1 expression whereas in Group-2 TREM-1 expression was minimal. SLX expression was similar in both the groups.

**Fig 10:**
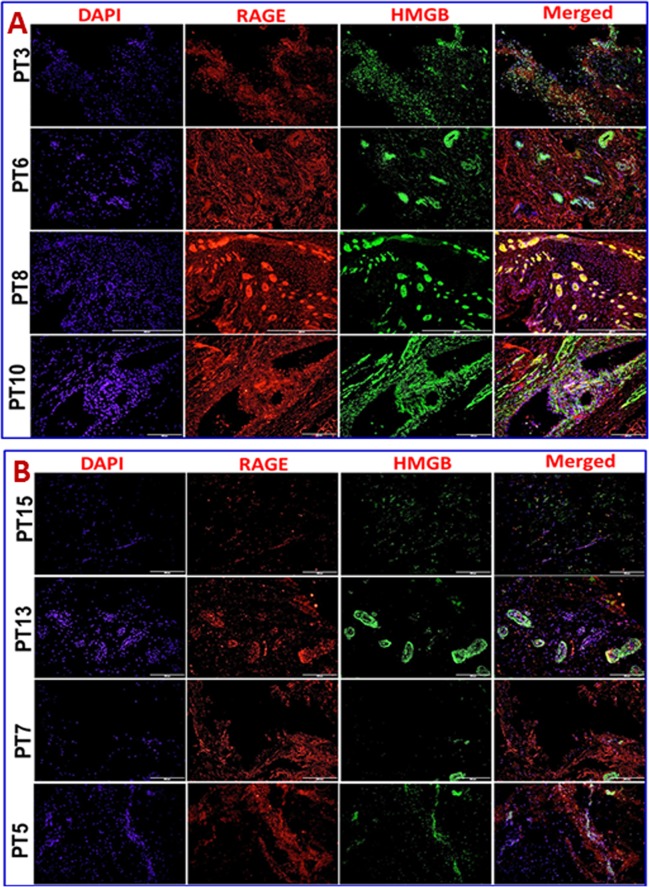
Double immunostaining for the co-localization of HMGB1 expression with RAGE on Group-1 (Patient numbers—PT3, PT6, PT8 and PT10) and Group-2 (Patient numbers–PT15, PT13, PT7 and PT5) tendons—(A) Group-1 and (B) Group-2 patients. Group-1 tendons have significantly higher expression of both HMGB1 and RAGE where in Group-2 both the proteins have lesser but prominent expression.

Immunofluorescence analysis was employed to assess the gene expression in tendon tissue specimen. Presence of CD68^+^ macrophages was prominent in Group-1 when compared with Group-2 (**Fig 4A and 4B**). Still, few of the Group-1 patients showed higher expression of CD68^+^ than others. Patients PT3 and PT8 of Group-1 showed more CD68^+^ macrophages, indicating the severity of chronic inflammation due to arthritis compared to the other two patients (PT6 and PT10) with arthritis. The evaluation of the fluorescence intensity further confirmed significant difference (**see Fig 5**). The fluorescence intensity for each sample was the average intensity of four different images taken from different areas of each specimen. Significant variation in the expression of CD68^+^ was observed between the groups. Similarly, TREM-1 expression was also found to be significantly higher in the tendon tissue of Group-1 patients (**Fig 5, Fig 6A and 6B)**. TREM-2 expression was higher in the tendon tissue of Group-1 compared to that in Group-2, but there were no significant differences between both groups (see **Fig 5**). Both TREM-1 and TREM-2 were found to be co-localized with tenomodulin (TM) (**Fig 6A and 6B, Fig 7A and 7B**) and scleraxis (SLX) (**Fig 8A and 8B, Fig 9A and 9B**) signifying TREM expression in tenocytes. The co-localization was more vivid in Group-2 (**Figs 6B, 7B, 8B and 9B**). The levels of SLX and TM expression were found to be insignificant between Group-1 and Group-2, but the images displayed relatively higher expression in Group-1, which could be due to the increased cell density (**Fig 5, Figs 6–9**). HMGB1 expression was significantly higher in Group-1 than that in Group-2 (**Fig 5, Fig 10A and 10B**). Similarly, RAGE was found to be significantly down-regulated in Group-2 in comparison to Group-(**Fig 5, Fig 10A and 10B**).

### Increased mRNA transcripts for TREM-1 but not TREM-2 in the shoulder tendon of arthritis compared to non-arthritis group

**Fig 11:**
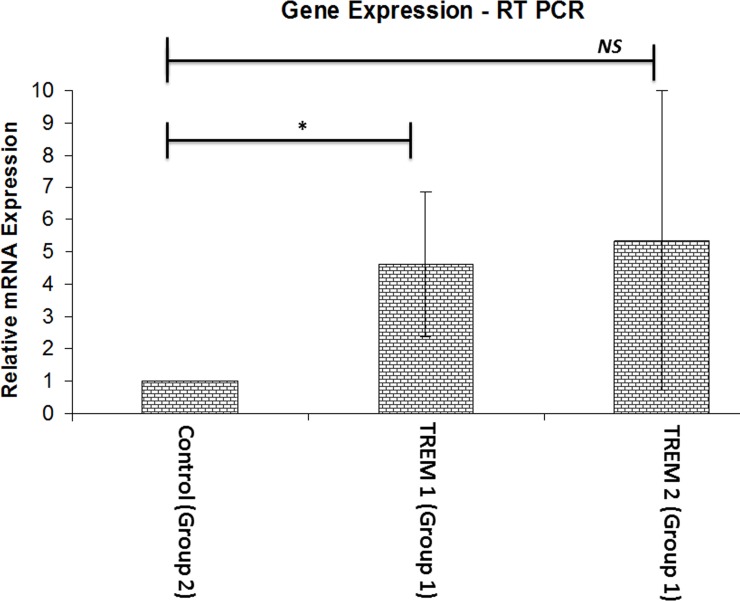
The mRNA expression of TREM-1 and TREM-2 genes in tendon tissue. The expression level of TREM-1 was significantly higher in Group-1 when compared to Group-2 as the control. There was no significant difference in TREM-2 expression between the Group 1 and Group 2. *Group-1 (n = 4)*, Group-1 (n = 8) * p<0.*05; NS–Not significant*.

The mRNA transcripts of TREM-1 and TREM-2 were observed in the tendon of both groups. There was about four-fold increase in the expression of TREM-1 in the tendon of Group-1 with respect to Group-2. TREM-2 expression was not significant (**Fig 11**). In regard to the inflammation associated with glenohumeral arthritis, we considered Group-2 (minimal inflammation) as a control for normalizing the TREM expression.

## Discussion

The exact mechanism underlying chronic inflammation of shoulder joint, rotator cuff tendon and associated muscles are largely unknown. The primary goal of the present study was to compare and correlate the expression levels of inflammatory mediator, TREM-1, and the major DAMP (damage associated molecular pattern) HMGB1 and its multi-ligand receptor RAGE with respect to patients with shoulder injury (Group-1) and without glenohumeral arthritis (Group-2). The principal findings in this study reveal significant expression of TREM-1, RAGE and HMGB1 in the shoulder tendon tissue with respect to severity of glenohumeral arthritis.

Severity of inflammation and ECM damage in Group-1 patients signifies the impact of glenohumeral arthritis in the healing response. Even though Group-2 patients had shoulder injury, the absence of arthritis/inflammation might have helped to restore ECM back to nearly normal (**Figs 1 and 2**). Chronic tendinopathy has been characterized by the absence of inflammatory cells in the load bearing regions of tendons [17–19]. Similar pattern was observed in the tissues of Group-2 patients where neutrophils and macrophages were rare. The prevalent morphologic alterations in the tendon tissue of Group-1 patients may be enhanced due to inflammation associated with glenohumeral arthritis. Fatty infiltration (in two cases; PT3 and PT10) and extreme tissue degeneration in Group-1 patients can be the impact of arthritis in shoulder tendinopathy [18].

Tendon tissue is mainly constituted by collagen matrix and tenocytes. The cell density of tenocytes was lower and the cells were usually appeared to be embedded in the collagen matrix. This was evident in the comparatively normal areas of Group-2 tendon sections which are characterized by the cells surrounding a matrix without disorganization. Clustering of tenocytes at the vicinity of disorganized matrix is considered to be one of the hallmarks of tendon pathology and was clearly evident in Group-2 patients. In order to cover maximum number of cells and major portion of tissue 20x magnification was employed for image acquisition after histology and immunofluorescence.

Tenocyte proliferation and metabolic activity are mediated by inflammatory cytokines (TGF-β) and growth factors. So, the colonization of tenocytes (hypertrophy and hyperplasia) can be an indirect evidence for inflammatory signaling [20]. Moreover, stresses like over load and hypoxia induce transformation of tenocytes to fibroblast like phenotype which may have implications on proliferation and growth factor signaling [9]. Thus, the repair response in the tendon tissue of Group-2 patients proceeds in an alternate manner than that in Group-1. Therefore, the status of inflammation could be one of the challenging factors for subsiding shoulder tendon injury.

The presence of CD68^+^ macrophages in Group-1 specimen implied an inflammatory phase imparted due to arthritis. Among these macrophages, M1 subtype (data not shown in this article) was predominant which reveals that Group-1 tendons were mostly in the inflammatory phase [21]. CD68^+^ macrophages were completely absent in the tendon tissue of Group-2 which signifies the possibility of alternate mode of inflammation mediated through tenocyte proliferation [22]. But, the signals that trigger such inflammatory responses are largely unknown. Published literature suggests that pain signaling molecule substance P acts on tenocytes to adopt a myofibroblast-like phenotype having higher smooth muscle actin expression and enhanced contractile properties. Such a transformed phenotype of tenocytes is evident in chronic painful tendinopathies. But, the mechanism of the activation of tenocytes and integration of multiple factors (cytokines, signaling molecules, transcription factors etc.) to elicit inflammatory response in tendons in the presence/absence of clinical conditions like arthritis warrants a detailed and careful investigation [2, 23].

Elevated levels of TREM-1 expression in the circulating cells and tendon tissue confirmed the inflammatory status of the tendon in Group-1 patients while comparing with Group-2. The role of TREM-1 in aggravating the acute and chronic inflammation has already been well established in both inflammatory and non-inflammatory diseases [7, 23–26]. TREM-1 is a member of immunoglobulin superfamily of receptors generally associated with neutrophils and monocytes/macrophages, and these receptors are triggered by as yet unidentified ligand(s). The transmembrane domain of TREM-1 is associated with an adaptor protein DAP12, which triggers the expression of inflammatory mediators like TNF-α, IL-8, monocyte chemotactic protein 1 (MCP-1) and others. A soluble form of TREM-1 (formed due to the proteolytic cleavage of membrane bound TREM-1 by matrix metalloproteases) also exist which is a well-known diagnostic marker for inflammation [27]. Increase in TREM-1 expression in peripheral blood neutrophil has been well documented for most arthritis types [28]. TREM-1 expression of CD16^+^ neutrophils was significantly greater in Group-1 patients (**Fig 3A and 3B**) and this can be attributed to the severity of arthritis. The expression of the anti-inflammatory TREM-2 was not prominent in the blood cells of both groups. However, there was a trend of higher density, but statistically insignificant, of TREM-1 and TREM-2 double-positive cells in the blood of Group-2 compared to Group-1 patients. The insignificant difference between the groups could be due to several factors, including variability among the patients in regard to unreported non-shoulder-related inflammatory condition and limited sample size.

TREM-1 expression was significantly elevated in both circulating cells and tendon tissue of Group-1 patients. This could be associated with arthritis, as evident from increased CD68^+^ macrophage infiltration [29]. Interestingly, Group-2 patients also displayed TREM-1 and TREM-2 expression where CD68^+^ macrophage infiltration was mostly absent. The histomorphology of Group-2 was also devoid of inflammatory cells. This led to the possibility that the cells of tendon tissue might express TREMs. This could be supported by our findings of increased mRNA transcription for TREM-1 and TREM-2 in the tendon tissue of Group-1 patients. This increased expression can be attributed to the increased cell number contributed by infiltrated CD68^+^ macrophage as a result of inflammation and arthritis. But, Group-2 also displayed considerable amount of mRNA transcription.

In order to examine if tenocytes possess the capability of TREM expression, we performed double immunostaining for TREMs and tendon specific biomarkers tenomodulin (TM) and scleraxis (SLX). Co-localization of TM and SLX with TREMs confirmed the presence of TREM-1/TREM-2 expressing tenocytes in the tendon (**Figs 6–9**). These findings suggest a role of TREM-1/TREM-2 expressing tenocytes in eliciting inflammatory response without the involvement of immune cells. No significant difference was observed in the expression of TM and SLX between both the groups, which implies a normalized expression.

To our knowledge, our findings are novel demonstrating TREM expression on tendon cells. But, human tissues like liver, heart, brain, placenta and kidneys have been found to express substantial levels of TREM-1 and TREM-2. However, the underlying mechanism of activation of these cells to secrete TREMs, their potential ligands, their functional role in these cells and the signaling cascade resulting in TREM gene expression are largely unknown [9, 30]. Group-2 patients also suffered from mild inflammation (which may be due to ECM disorganization) and pain. Expression of TREM-1 in these patients can be a limiting factor for sustenance of ECM damage and delay in healing and pain. In Group-1 patients the classic inflammatory pathways will be executed by the mediation of inflammatory cells like neutrophils and macrophages [31].

HMGB1 is a nonhistone DNA binding protein which is loosely associated with chromatin. In cells under stress, HMGB1 translocates to cytosol and reaches ECM if plasma membrane integrity is lost. So, the possibility of HMGB1 release in necrotic and/or apoptotic cells is very high. Released HMGB1 is considered to be a DAMP that stimulates innate immune receptors and triggers inflammation [32]. HMGB1 was reported to induce cytokine expression, facilitate cell migration and chemotaxis, activate immune cells and non-immune cells like fibroblasts and endothelial cells, and stimulate antibody production. Moreover, HMGB1 is confined to local sites of inflammation and their stimulation requires higher concentration than conventional cytokines [33]. HMGB1 also induces the classic inflammatory reactions even in sterile injury/ischemic injury [34]. Among the signals (IFN-γ, TNF-α, IL-1β and NF-κβ) that trigger HMGB1 secretion, the multi-ligand receptor RAGE, has gained significance due to its potential role in inflammation. Apart from DAMPs like HMGB1, RAGE also binds with advanced glycation end products (AGEs) and RAGE activation plays a significant role in the progression of inflammatory diseases like arthritis [35].

The expression of HMGB1 and RAGE in the tendon tissue of Group-1 patients was significantly higher than in Group-2 (**Fig 10A and 10B**). This can be attributed to the presence of inflammatory cells. Interaction of HMGB1 with RAGE in inflammatory cells triggers cell migration and angiogenesis resulting in the recruitment of more inflammatory cells and release of mediators. At the same time HMGB1 interferes with phagocytosis which results in sustained inflammation. Failure of phagocytic removal of apoptotic cells paves way for tissue necrosis that in turn fuels inflammation [35]. This could be a reason for increased expression of HMGB1 and RAGE and angiogenesis in the tendon of Group-1 patients. On the other hand, tendon tissue in Group-2 also displayed considerable amount of HMGB1 and RAGE even in the absence of inflammatory cells. This may be due to ischemia/necrosis-mediated sterile inflammation and stress associated with the injury. The exact mechanisms of HMGB1 and RAGE activation and their functions in tendons in relation to sterile inflammation are yet to be unveiled [22].

Thus, we are the first to report TREM-1 and TREM-2 expression in tendon tissues and their participation in inflammatory responses. The increased levels of TREM-1, RAGE and HMGB1 expression and inflammatory status in both the group represent interrelationship among these mediators. These mediators were active upon encounter with the inflammatory cells as well as on resident tenocytes. HMGB1 functions as an activating ligand for TREM-1 in various cell type (immune as well as non-immune cells) and the binding was reported to be concentration- dependent [36, 37]. Still the underlying mechanism, signals triggering their activation and binding, their clearance after signaling and their potential role in healing responses in tendon tissues are yet to be resolved. The concomitant increase in the expression of TREM-1, RAGE and HMGB1 with respect to inflammation associated with glenohumeral arthritis and sterile inflammation signifies the involvement of these molecules in tendinopathy.

### Potential Limitations

Limited availability of tissue specimen, lack of normal control, difference in age and variations in physiological and pathological status of the patients are the major limitation of this study. The expression of TREM1, HMGB1 and RAGE due to inflammation from the causes other than shoulder arthritis cannot be ruled out, even though there was no report of any obvious inflammation in the organs other than the shoulder of the patients. None-the-less, the elucidation of biology of these molecules can open new opportunities in the therapeutic regimes for musculoskeletal diseases like shoulder injury.

## Materials and Methods

### Blood and tissue collection and processing

The study was approved by the Creighton University Institutional Review Board. The study protocol and the details of the procedures were explained to the participants in layman terms, and the written informed consent and the HIPPA forms were signed by the volunteers prior to the surgery for collection of tissue and blood specimen.

Fifteen patients having RCI (40–65 years old) were recruited in the study; four of them were suffered from glenohumeroid arthritis. The remaining eleven patients had RCI and but devoid of glenohumeroid arthritis. All the patients were undergone shoulder surgery and biceps tendons were harvested. All the patients complained about shoulder pain and exhibited classical symptoms of inflammation. The tendon tissue and blood samples from the patients were numbered as PT1 to PT15 and PB1 to PB15, respectively. The findings from the MRI of the shoulders were used to categorize the patients with respect to the severity of arthritis and inflammation. The patients were classified into two groups; Group-1 includes 4 patients (PT3, PT6, PT8 and PT10) with severe glenohumeroid arthritis and rotator cuff tear (superior labral anterior to posterior (SLAP)), and Group-2 consists of the remaining 11 patients with rotator cuff pathology (SLAP) and without arthritis. Rotator cuff tears were confirmed intraoperatively during routine shoulder arthroscopy. A subpectoral biceps tenodesis was performed in all patients for clinically symptomatic bicipital symptoms, and the intra-articular portion of the biceps was resected and obtained for tissue processing. The biceps specimens were collected in the UW (University of Wisconsin) solution for temporary storage and transportation. The proximal portions of the specimen were taken for analysis. Blood (8 mL) was also drawn in citrated tubes from each patient using a butterfly cannula and processed immediately.

### Histology

The specimens were fixed in 10% formalin for 24 h and embedded in paraffin wax and heat-fixed on microscopic slides. Tissue sections of 5μm thickness were cut using microtome (Leica, Germany) along the horizontal axis [38]. The sections were then deparaffinized with xylene and dehydrated with graded concentrations of ethanol and used for Hematoxylin and Eosin (H&E) staining [39]. Xylene-based mounting media was used for mounting the stained sections. The slides were imaged using an inverted microscope attached with CCD camera (Olympus BX51; Olympus America, Center Valley, PA) under bright field mode [23]. The histological evaluation of slides was performed qualitatively and independently by two blinded investigators which were confirmed by the third one.

### Flow cytometry

Expression of TREM-1 and TREM-2 in peripheral blood neutrophils and macrophages was determined by flow cytometry. Blood (100μl) from the patients was incubated for 30 min at room temperature in dark with anti-human FITC-conjugated CD45, PE-Cyanine-5-CD16, Allophycocyanin-CD14, PerCP-Cy5.5-HLA-DR, PE-TREM-1 and APC-TREM-2. After incubation the RBCs were lysed by adding FACS lysing solution (eBioscience, San Diego, USA) and the WBCs were pelleted by centrifugation at 500g for 5 min. The cells were fixed with FACS fixing solution (4% formalin) and the expression of markers was analyzed by FACS analyzer (BD biosciences). Isotype controls (isotypes instead of antibodies) and untreated blood as a control were also run in parallel. Neutrophils and monocytes/macrophages were gated and the analysis of TREM-1 and TREM-2 expression was performed using FlowJo single cell analysis software.

### Immunofluorescence

The deparaffinized sections were subjected to antigen retrieval in HIER buffer (Heat Induced Epitope Retrieval) at 95°C for 20 min. The sections were then washed in PBS and incubated with blocking solution containing 0.25% Triton X-100 and 5% horse serum in PBS for 2h at room temperature. After blocking the slides were allowed to bind with 1:50 diluted primary antibodies for 4°C overnight. For dual staining cocktail of two antibodies and for monostaining corresponding individual antibodies were used. All the antibodies were purchased from SantaCruz Biotech. The unreacted primary antibodies were washed in PBS and fluorochrome-conjugated corresponding secondary antibody (1:100 dilution) was added and allowed to bind with the primary antibody for 2h at room temperature. After washing with PBS the nuclei were counterstained with 4′,6- diamidino-2-phenylindole (DAPI)-containing mounting media. The sections were visualized under a fluorescent microscope (Olympus BX51; Olympus America, Center Valley, PA). The images were acquired using Olympus DP71 camera and automatically merged using the software associated with the camera. Three or four images were represented in the result section and the fluorescence intensity corresponding to gene expression was assessed by ImageJ software. For intensity calculation by ImageJ, four images of each patient taken from four different sections were analyzed and their average intensity was considered for statistical analysis. A negative control (secondary antibody alone) was maintained [19, 40]. The primary antibodies used in the study include: mouse anti-human CD-68, rabbit anti-human TREM-1, rabbit anti-human TREM-2, mouse anti-human HMGB-1, goat anti-human RAGE, goat anti-human tenomodulin (TM) and goat anti-human scleraxis (SLX). The combination cocktail consisted of TREM-1 and tenomodulin, TREM-2 and tenomodulin, TREM-1 and scleraxis, TREM-2 and scleraxis, and HMGB-1 and RAGE. The secondary antibodies used were either FITC- or Texas red-conjugated with respect to the primary antibody (donkey anti-mouse, goat anti rabbit and rabbit anti-goat).

### Real time PCR

#### RNA isolation from tendon

About 200mg tissue taken from the proximal portion of the biceps tendon was minced to small pieces in 250μl UW solution. Trizol reagent (1ml) was added to the tissue followed by homogenization and vortex and then kept at room temperature for 10 min. Chloroform-isoamyl alcohol reagent (200μl) was added, centrifuged at 12,000 rpm for 15 min. To the aqueous layer, 500μl isopropanol was added, kept for 10 min at room temperature to precipitate RNA, and centrifuged at 12,000 rpm for 10 min to pellet down the RNA. The pellet was washed with 75% ethanol, dissolved in sterile RNase free water, quantified and stored at -80°C [22].

#### PCR

Total RNA (1μg) from tendon tissue was reverse transcribed to cDNA using PROMEGA cDNA synthesis kit according to the manufacturer’s protocol. The RNA and oligo-dT primers were primed prior to the reaction. Reaction mixture (MgCl_2_, reaction buffer, dNTP mix, RNase inhibitor and reverse transcriptase) was prepared for 20μl reaction volume and cDNA synthesis was carried out in Bio-Rad T100 thermal cycler. Gene expression levels of TREM-1 and TREM-2 were quantified by the real-time PCR system (Bio-Rad T1000 thermal cycler). The 8μl cDNA (1:1 dilution) was mixed with 10μl SYBR Green PCR master mix (PROMEGA) and corresponding forward and reverse primers (10pM/μl) (**Table 1**) for real-time PCR amplification. The 18s rRNA was used as the housekeeping gene. The reaction conditions were 95°C for 10 minutes, 40 cycles at 95°C for15s, and 60°C for 1 min. The mRNA expression of TREM-1 and TREM-2 was normalized with the expression level of 18s rRNA. Four independent experiments were carried out for each sample and average of Cq values was taken. Results are represented as fold-change of TREM-1 and TREM-2 mRNA levels between patients and the reference gene [41].

**Table 1:** Primers and their sequences used for RT-PCR analysis.

Primers	Sequence
TREM-1 Forward	5’AGTTACAGCCCAAAACATGC3’
TREM-1 Reverse	5’CAGCCCCCACAAGAGAATTA3’
TREM-2 Forward	5’ACAGAAGCCAGGGACACATC3’
TREM-2 Reverse	5’CCTCCCATCATCTTCCTTCA3’
18s Forward	5’TCAACTTTCGATGGTAGTCGCCGT3’
18s Reverse	5’TCCTTGGATGTGGTAGCCGTTTCT3’

### Statistical analysis

The results are presented as mean ± SEM (standard error of the mean) and the statistical significance was analyzed using Mann-Whitney U test for non-parametric data. The level of significance was set at *p <0*.*05*. Values that were extremely far from the mean were excluded.

## Conclusions

The expression of TREM-1, RAGE and HMGB1 in shoulder tendon tissue was significant with respect to severity of glenohumeral arthritis. The same trend was also observed for TREM-1 in circulating neutrophils. The infiltration of macrophages was significantly high in Group-1 while it was mostly absent in Group-2. TREM-1 and TREM-2 expression in the nearly normal tendons of Group-2 patients, even in the absence of immune cells, revealed TREM expression by tenocytes which was confirmed by double immunostaining with tendon-specific biomarkers like TM and SLX. This showed the persistence of mild inflammation in Group-2 patients which proceeds without the involvement of inflammatory cells. The DAMP molecule, HMGB1, was highly expressed in Group-1 which corresponds to inflammation while in Group-2 HMGB1 expression was significantly lower than the Gorup-1, and this could have implications for asymptomatic tendon injury. Overall, the findings suggest that HMGB1-mediated TREM-1 activation can occur via alternate routes depending on the severity of inflammation and the presence/absence of inflammatory cells.
